# Impact of Mucin on Drug Diffusion: Development of a Straightforward In Vitro Method for the Determination of Drug Diffusivity in the Presence of Mucin

**DOI:** 10.3390/pharmaceutics12020168

**Published:** 2020-02-17

**Authors:** Margherita Falavigna, Paul C. Stein, Gøril Eide Flaten, Massimiliano Pio di Cagno

**Affiliations:** 1 Drug Transport and Delivery Research Group, Department of Pharmacy, UiT The Arctic University of Norway, Universitetsvegen 57, 9037 Tromsø, Norway; margherita.falavigna@uit.no (M.F.); goril.flaten@uit.no (G.E.F.); 2 Department of Physics, Chemistry & Pharmacy, University of Southern Denmark, Campusvej 55, 5230 Odense, Denmark; pcs@sdu.dk; 3 Site-specific Drug Delivery Group, Department of Pharmacy, Faculty of Mathematics and Natural Sciences, University of Oslo, Sem Sælands vei 3, 0371 Oslo, Norway

**Keywords:** mucosal drug delivery, mucin, mucin–drug interaction, diffusion, UV-visible localized spectroscopy, permeability

## Abstract

Mucosal drug delivery accounts for various administration routes (i.e., oral, vaginal, ocular, pulmonary, etc.) and offers a vast surface for the permeation of drugs. However, the mucus layer which shields and lubricates all mucosal tissues can compromise drugs from reaching the epithelial site, thus affecting their absorption and therapeutic effect. Therefore, the effect of the mucus layer on drug absorption has to be evaluated early in the drug-development phase, prior to in vivo studies. For this reason, we developed a simple, cost-effective and reproducible method employing UV-visible localized spectroscopy for the assessment of the interaction between mucin and drugs with different physicochemical characteristics. The mucin–drug interaction was investigated by measuring the drug relative diffusivity (D_rel_) in the presence of mucin, and the method was validated by fitting experimental and mathematical data. In vitro permeability studies were also performed using the mucus-covered artificial permeation barrier (mucus–PVPA, Phospholipid Vesicle-based Permeation Assay) for comparison. The obtained results showed that the diffusion of drugs was hampered by the presence of mucin, especially at higher concentrations. This novel method proved to be suitable for the investigation on the extent of mucin–drug interaction and can be successfully used to assess the impact that the mucus layer has on drug absorption.

## 1. Introduction

Over the past decades, mucosal drug delivery has received increasing attention both for systemic and local drug administration [1]. This strategy comprises not only the oral administration route, but also the buccal, nasal, pulmonary, ocular, rectal, and vaginal ones, offering several different sites and a large surface area suitable for drug absorption [2]. The bioavailability of drugs after mucosal absorption depends on numerous and dynamic interactions occurring even before the direct contact of the drug with the mucosal epithelium. In particular, the mucus layer, which covers, lubricates and protects all mucosal epithelial surfaces, is one of the first barriers that drugs and formulations have to overcome before reaching their site of action and explicate their effect [3]. Therefore, it has become crucial to be able to study the interaction between the mucus layer and drugs to best assess their drugability and in order to develop formulations able to specifically target this layer.

Mucus is mainly composed of water (95%), glycoproteins and lipids (0.5–5%), mineral salts (0.5–1%) and proteins (1%). The glycoproteins contained in the mucus layer (i.e., mucins) are composed of a hydrophobic peptide backbone linked to multiple hydrophilic oligosaccharide chains, forming a bottle brush conformation [3]. Mucins are the main determinant of the gel-like rheology of mucus [4], and their highly glycosylated regions give mucus an overall hydrophilic behavior, with a distinctive negative charge caused by the prevalence of sialic acid in the terminal part of the oligosaccharide chain [1].

The mucus layer is able to act as a barrier for the diffusion of foreign entities, such as drugs and particles [5]. For instance, lipophilic drugs proved to have particular affinity to the non-glycosylated regions of mucins, thus their diffusion through the mucus layer would be slowed down more than hydrophilic ones. On the other hand, positively charged drugs/particles can electrostatically bind the negatively charged mucins, and this interaction can cause their retention in the mucus layer and slow down their diffusion through it [4].

In the past decades, several different techniques have been established to study the interplay between drugs/formulations and the mucus layer, such as diffusion studies [6,7], microscopy-based techniques [8], SANS (small-angle neutron scattering) [9], NMR (nuclear magnetic resonance) methodology [10], together with in vivo [8] and in vitro models [11].

However, most of these techniques rely on complex and costly apparatuses, resulting in a poor applicability for those laboratories that do not have access to the specific instruments. Moreover, a physical parameterization of drug diffusion through mucin is severely lacking. Thus, in the present study we investigated the applicability of a previously established method based on UV-visible localized spectroscopy [12] for the assessment of drug–mucin interaction, in order to experimentally quantify the relative diffusivity (D_rel_) of drugs in mucin. This versatile and easy-to-implement method allows the measurement of very precise diffusivities (D, experimental error below 5% and fitting error below 1% [12]) for all chemical entities containing a chromophore by employing a standard UV-visible spectrophotometer. Empirical data are then analyzed by standard non-linear data fitting procedure in order to find diffusivity values. This method previously proved to be efficient in order to study complex systems such as drug-cyclodextrin interactions and drug-liposomes interaction [13]. Thus, in this work this method has been applied to investigate drug–mucin interactions.

## 2. Materials and Methods

### 2.1. Materials

Atenolol (ATN), caffeine (CAF), chloroform, ethanol (96%, *v/v*), hydrocortisone (HYD), methanol CHROMASOLV^®^, mucin from porcine stomach type III (MUC) (bound sialic acid 0.5–1.5%, partially purified), naproxen (NPR), potassium phosphate monobasic (KH_2_PO_4_), sodium chloride (NaCl), sodium hydroxide (NaOH), sodium phosphate dibasic dihydrate (Na_2_HPO_4_·2H_2_O), sodium phosphate dibasic dodecahydrate (Na_2_HPO_4_·12H_2_O), sodium phosphate monobasic monohydrate (NaH_2_PO4·H_2_O) were products of Sigma-Aldrich Chemie GmbH (Steinheim, Germany). E80 lipoid egg-phospholipids (80% phosphatidylcholine) were obtained from Lipoid GmbH (Ludwigshafen, Germany). All chemicals employed were of analytical grade. For the preparation of the PVPA (Phospholipid Vesicle-based Permeation Assay) barriers, nucleopore track-etch membrane filters (0.4 and 0.8 μm pore size) were purchased from Whatman (part of GE Healthcare, Oslo, Norway) and the nitrocellulose membrane filters (0.65 μm DAWP) were obtained from Millipore (Billerica, MA, USA). Transwell filter inserts and plates (d = 6.5 mm) were products of Corning Inc. (Corning, NY, USA).

### 2.2. UV-Visible Localized Spectroscopy

#### 2.2.1. Drug Solutions Preparations

Phosphate buffered saline (PBS) 65 mOsm/kg (pH 7.4) was prepared following the procedure previously described by Wu et al., (2017) [14]. Briefly, NaH_2_PO_4_·H_2_O, Na_2_HPO_4_·2H_2_O, NaOH, and NaCl (4.5 g/L, 7.4 g/L, 0.8 g/L, and 4.4 g/L, respectively) were dissolved in distilled water to obtain a 300 mOsm/kg buffer (pH 7.4). The buffer was diluted 1:5 (*v/v*) with distilled water to obtain the desired ionic strength (PBS 65 mOsm/kg; pH 7.4). The osmolality of the final buffer was measured using a Semi-Micro Osmometer K-7400 (Knauer, Berlin, Germany), whereas the pH was checked with the sensION^TM^ + PH31 pH meter (Hach, Barcelona, Spain). ATN, CAF, HYD, and NPR were dissolved in PBS 65 mOsm/kg (pH 7.4) to achieve a final nominal concentration (C_n_) of 5, 0.86, 0.53, and 1.01 mM, respectively.

#### 2.2.2. Mucin–Drug Samples Preparation

Mucin type III from porcine stomach was dispersed in PBS 65 mOsm/kg (pH 7.4) to obtain 0.1, 0.3 and 0.6% (*w/w*) mucin dispersions. ATN, CAF, HYD and NPR were dissolved in the mucin dispersions (0.1, 0.3, and 0.6% *w/w*) to achieve the same final concentration described in Section 2.2.1.

#### 2.2.3. Analytical Method

The analytical method developed by di Cagno et al., (2018) [12] was employed to study the influence of mucin on the passive diffusion of drugs with different physicochemical characteristics (Table 1). Briefly, a conventional double beam VWR UV-6300 PC UV-visible spectrophotometer (VWR International, Radnor, PA, USA) was utilized, together with two Hellma^®^ Suprasil^®^ quartz absorption cuvettes with a chamber volume of 700 µL and path length of 10 mm (Sigma-Aldrich, Steinheim, Germany). Then 675 µL of distilled water were pipetted into the two cuvettes (reference and sample cuvette), and placed in the adequate compartment of the spectrophotometer. At the start of the experiment (t = 0 s) and with the aid of a needle syringe, 25 µL of the (mucin-) drug sample were carefully injected in the bottom of the sample cuvette. Once the experiment had started, both cuvettes were closed with parafilm to avoid evaporation of their content. Drug diffusion was followed by measuring the absorbance of each drug at 0.51 cm from the bottom of the cuvette (h_m_). The detection of the absorbance for the different drugs was recorded at room temperature every 120 **s** for a total of 18 h (23–25 °C), at 273, 272, 247, and 270 nm for ATN, CAF, HYD, and NPR, respectively. The diffusion of mucin (MUC 0.1, 0.3, and 0.6% *w/w*) in water was also investigated at 247 and 272 nm, in order to assess its impact on the diffusion of the mucin–drug sample. Prior to the establishment of this experimental method, the diffusion of drugs dissolved in aqueous solution (25 µL) through a mucin dispersion (675 µL) was also evaluated. However, the high variation in diffusion curves pointed out a reliability issue connected with the described setup, thus the one where the drug was dissolved in mucin and diffusing in water was solely applied.

Experimental data were treated according to di Cagno et al., 2018 [12]. In brief, Fick’s second law of diffusion was analytically solved (Equation (1)) assuming times (*t*) and positions (*x*) such that *t* ≪ h2/D and *x* ≪ h (where h is the full length of the cuvette occupied by water, 3.30 cm). Experimental data were fitted (MatLab program, MathWork Inc., Natick, USA) to Equation (1) in order to obtain σ (the width of the initial distribution considered to be a half Gaussian curve [12]), A (the initial amount of the API) and D (diffusion coefficient).

(1) $$c\left(x,t\right)=\frac{2A}{\sqrt{\pi }}\;\frac{e^{\frac{-x^{2}}{2\sigma^{2}+4Dt}}}{\sqrt{2\sigma^{2}+4Dt}}$$

Mass preservation is assumed through the whole experiment such as:$\;\int_{0}^{\infty }dxc\left(x,t\right)=A$.

In this article we will use A_0_ and D_0_ to indicate the parameters measured in pure water (i.e., without mucin).

### 2.3. Mucus–PVPA Barrier Preparation

The impact of mucin on drug permeability was investigated following the method described by Falavigna et al. (2018) [20]. Briefly, the PVPA barriers were prepared by depositing liposomes with different sizes (diameter 0.4 and 0.8 µm) on top of nitrocellulose membrane filters by centrifugation, followed by freeze-thaw cycles to provide immobilization and fusion of the liposomes in and on top of the membrane filters. To assess the effect that the mucus layer has on drug permeability, a mucin dispersion (mucin type III from porcine stomach, 1.0% *w/w* in PBS pH 7.4) was prepared, pipetted on top of the PVPA barriers (50 µL) and left to incubate for 5 min at room temperature prior to the start of the permeability study.

### 2.4. In Vitro Permeability Study

The permeability of ATN, CAF, HYD, and NPR was studied both in the presence and absence of the mucin layer using the mucus–PVPA model (Falavigna et al., 2018) [20]. In the case of the experiment carried out in the presence of mucin, the mucin dispersion was pipetted (50 µL) on top of the PVPA barriers and left to incubate for 5 min prior to the addition of the drug solution. The drug solution was then pipetted (100 µL) on top of the PVPA/mucus–PVPA barriers, and the inserts which constitute the donor compartment (mucus–PVPA barriers + drug solution) were subsequently placed in wells containing the acceptor medium (600 µL of PBS pH 7.4). To maintain sink conditions, the inserts were positioned in new acceptor compartments containing fresh acceptor medium after 1, 2, 3, 3.5, 4, 4.5, and 5 h. Moreover, the concentrations of drug solutions placed in the donor compartment were chosen in order to ensure that the solubility limit in the acceptor compartment would not be reached. At the end of the permeability study, the amount of drug permeated in the acceptor compartment was quantified spectrophotometrically using a SpectraMax 190 Microplate reader (Molecular Devices Corporation, San Jose, CA, USA). ATN, CAF, HYD, and NPR were quantified at 273, 272, 247, and 270 nm, respectively. Moreover, the electrical resistance across the PVPA barriers was measured to confirm their integrity and proper functionality, as previously described [20,21,22,23]. The P_app_ (apparent permeability, cm/sec) of the investigated drugs was calculated by Equation (2).

(2) $$P_{app}=\frac{dQ}{dt}\times \frac{1}{S\times Cd}$$

where *dQ/dt* (nmol/s) is the slope at the steady state conditions, *Cd* (nmol/mL) the concentration of the drug in the donor compartment and *S* (cm^2^) the surface area of the PVPA barriers (0.33 cm^2^). The permeability experiment was conducted in triplicate for each drug with 6 PVPA barriers in each replicate.

### 2.5. Statistical Analysis

The statistical analysis of the permeability results described in Section 2.4 was performed with GraphPad Prism 7.03. Student *t*-test was utilized to highlight significant differences (*p* < 0.05) between the permeation of drugs in the presence and absence of mucin.

## 3. Results and Discussion

### 3.1. Quantification of Diffusion Coefficients in Water (D_0_)

**Figure 1:**
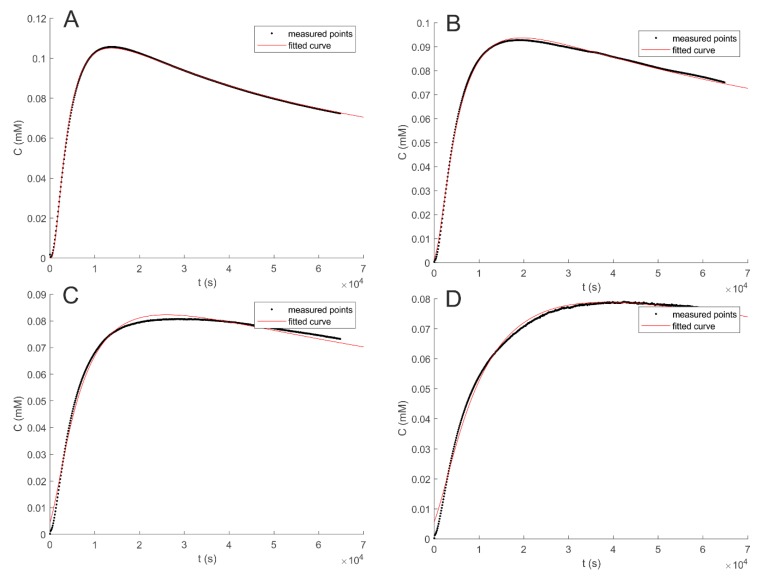
Diffusion profiles of caffeine (CAF) in water (**A**), MUC 0.1 (**B**), 0.3 (**C**), and 0.6% (**D**) (*w/w*). Absorbance recording point was fixed at 0.51 cm from the origin of the diffusion.

The diffusion coefficient of ATN, CAF, HYD, and NPR in water (D_0_) were obtained by fitting the experimental data to Equation (1) with MatLab. The results are summarized in Table 2. Firstly, as it can be observed in Figure 1 (and Figures S1–S3 in Supplementary Materials), the fitting of the experimental (black points) and the mathematical curve (red line) was found to be in accordance with previous work [12] both in the presence and absence of mucin.

The satisfactory correspondence between experimental data and the fit was also confirmed by the correlation between the nominal injected concentration (C_n_) and the initial amount of drug (A_0_) (*R^2^* = 0.9998).

The Stokes–Einstein equation (Equation (3)) describes the relation between the diffusion constant (D) and the size of a hypothetical spherical particle with radius r:

(3) $$D=\frac{k_{B}T}{6\pi \eta r}$$

Other relevant parameters in the relation between D and r of a spherical particles are temperature (T), Boltzmann constant (k_B_), and viscosity of the diffusional media (η).

**Figure 2:**
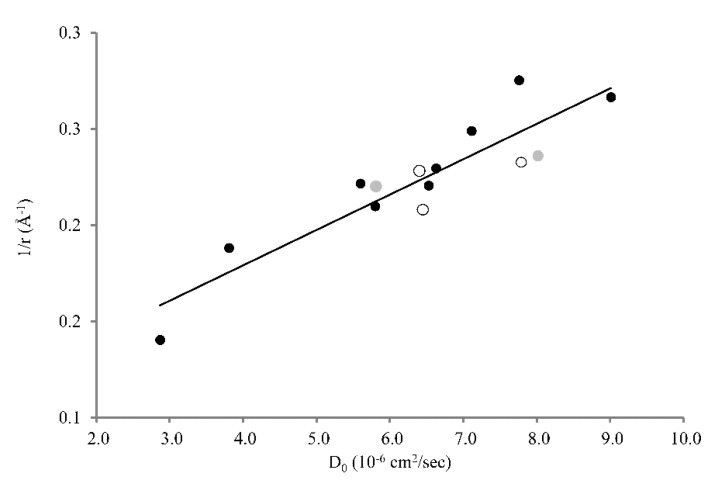
Correlation between D_0_ of 14 compounds and their estimated molecular radii (r) (D_0_ vs. 1/r). Empty dots represent the compounds measured in this work whereas grey and the black dots correspond to data taken from the literature [12,13].

Assuming that: i) all experiments are carried out at the same temperature, ii) the viscosity is not affected by the concentration of the diffusing drug, and iii) that the drugs’ hydration shells do not depend on mucus concentration, we can assume that D should be proportional to 1/r for the investigated drugs (at least for small molecules, where the structural approximation to a sphere might hold). By plotting the diffusion constants found in di Cagno et al., (2018) [12], di Cagno and Stein (2019) [13], and in the present study against the molecular radii of the examined drugs, an acceptable linear correlation between D and 1/r is found (*R^2^* = 0.8; Figure 2). In particular, the diffusion constants of larger molecules are found to be lower (e.g., HYD, D_0_ = 6.44 × 10^−6^ cm^2^/s) compared to smaller ones (e.g., caffeine, D_0_ = 9.07 × 10^−6^ cm^2^/s) (Table 1, Table 2).

### 3.2. Quantification of Relative Diffusion Coefficients (D_rel_)

**Figure 3:**
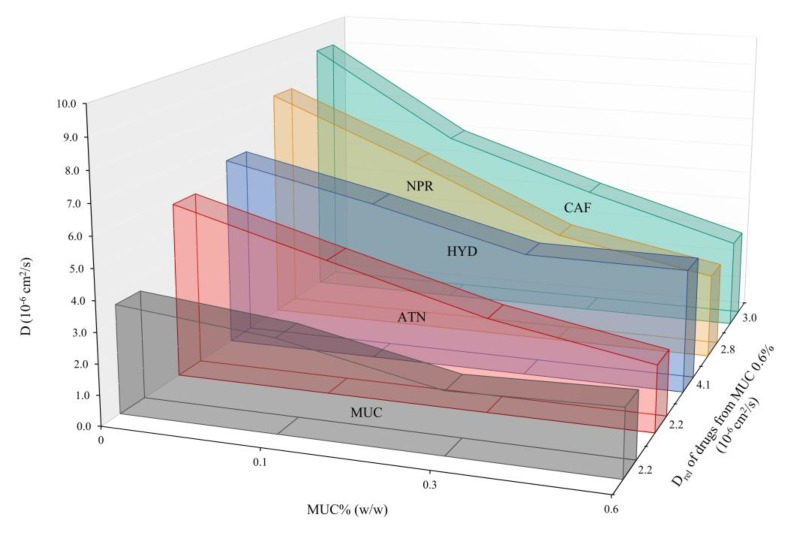
Relation between drug diffusivities (D) and the different mucin concentrations (MUC 0.0, 0.1, 0.3, and 0.6% *w/w*) for ATN, CAF, HYD, NPR, and MUC. Data reported represents the average of two parallel experiments (variation below 5% and fitting error below 1%).

Figure 3 displays the diffusion of the investigated drugs (ATN, CAF, HYD, and NPR) from mucin dispersion at different concentrations (MUC 0–0.6% *w/w*), together with the diffusion of mucin (MUC) itself in water. As it can be observed from Figure 3, mucin was able to diffuse in water with decreasing diffusivities following its increase in concentration. In fact, diffusivity of mucin was found to be 3.1 and 2.2 × 10^−6^ cm^2^/s for MUC 0.1 and 0.6% *w/w* at 272 nm, respectively. No significant differences were observed when absorbances where recorded at 247 nm. This significant reduction in diffusivity could be due to changes in viscosity of the donor solution, as a more viscous mucin dispersion would diffuse slower than a less viscous one. As it can be observed in Figure 3, D of mucin was found to be lower than all investigated drugs. Thus, the method used in this study allows the investigation on the diffusivities of both drugs and macromolecules.

Results showed that D_rel_ (i.e., the diffusivities of drugs in the presence of mucin) differs from D_0_ (i.e., the drug diffusivity measured in water only) for all drugs investigated and that the magnitude of these differences was highly dependent on mucin concentration in the donor solution. As it can be seen in Figure 3, D_rel_ appears to decrease with increased mucin concentration for all drugs investigated. This is to be expected as higher mucin concentration implies higher viscosity (η) and reduced D (Stokes–Einstein equation, Equation (3)). The percentage reduction of D_rel_ in comparison to D_0_ (i.e., no mucin) at higher concentration of mucin ranged within 63% for ATN, 65% for NPR and 67% for CAF (Table 3). Interestingly, HYD was less influenced by the presence of mucin, with a maximum reduction of D of 37% at the highest concentration of mucin (MUC 0.6%) in comparison to D_0_ (Table 3). Assuming a linear dependence between viscosity and mucin concentration, and that the drugs’ hydration shells do not depend on mucus concentration, a linear relationship should also be expected (according to Stockes law, Equation (3)) between mucin concentration (i.e., viscosity, η) and 1/D_rel_. Indeed, this is the case for ATN (*R^2^*= 0.999), NPR (*R^2^*= 0.987) and CAF (*R^2^*= 0.988), indicating a strong influence of viscosity on the reduction of D_rel_ for these three drugs. Also for HYD, a significant decreasing in D_rel_ was observed, indicating that viscosity is also affecting this compound and specifically its ability in diffusing through mucin. However, HYD seems to have a different behavior (*R*^2^ = 0.734) and the reduction in D_rel_ was found to be smaller than for the other compounds. Due to the experimental set-up chosen (i.e., mucin just in the donor), it is not possible to rule out the eventual impact of mucin absorbance on the net diffusion profiles of the drugs. However, this should be relevant just at higher mucin concentrations (above 0.3%). In any case, the eventual variance should be considered as a systematic error, as mucin concentrations were the same for all drugs investigated, therefore allowing internal data comparison. Moreover, on top of the effect that increased viscosity can have, additional compound-specific interactions (i.e., size filtering, interaction filtering and pH-dependent water solubility) can take place between drug and mucin, and can thus have an effect on drug diffusivity.

In fact, the degree of hindrance of the mucus layer towards the diffusion/permeation of drugs is particularly dependent on two main mechanisms: interaction and size filtering [5]. Interaction filtering accounts for those hindrance events that are caused by electrostatic/hydrophobic interactions, hydrogen bonds, and specific binding interactions between mucus and the investigated drug, whereas size filtering depends on the mucus mesh spacing, which can prevent the diffusion of larger molecules [5]. For instance, it has been previously shown that the diffusion of small and uncharged drugs is slowed down less by the mucus layer compared to charged or larger ones [3]. Indeed, mucins display highly glycosylated regions, which translate into their overall hydrophilic nature, and the prevalence of sialic acid in the terminal part of the oligosaccharide chain give mucins their distinct negative charge [1]. These characteristics allow electrostatic interactions with hydrophilic and positively charged drugs, which lead to mucin binding [4]. This can be clearly observed in the case of the diffusion of the positively charged ATN (Table 1) in MUC 0.6%, as the D_rel_ of the drug is very close to the one of MUC 0.6% itself (2.2 cm^2^/s), suggesting that a considerable amount of ATN is diffusing while being bound to mucin. In the case of CAF, the diffusion from mucin was slowed down to a similar extent compared to ATN (Table 3, Figure 3). Even though quite lipophilic, NPR seems to behave quite similarly to the two hydrophilic compounds (ATN and CAF), showing a similar trend in reduction of D with increased mucin concentration (Figure 3). Nevertheless, this behavior is not unexpected as NPR at pH 7.4 is majorly ionized due to the dissociation of the carboxylic group (pKa of 4.2; Table 1), making this compound more hydrophilic in neutral/basic conditions. On the other hand, the non-glycosilated regions of mucin show affinity for lipophilic drugs [4], allowing mucin binding for this class of compounds as well. In this study, these interactions seem to be of lower magnitude in comparison to the hydrophilic ones (e.g., ionic bond, hydrogen bond formation, etc.). This is not very surprising as hydrophilic interactions such as hydrogen bonds and ionic bonds are much stronger as well as of shorter-range than non-polar interactions such as Van der Waals forces. In fact, the reduction of D_rel_ in the case of HYD (neutral lipophilic compound; Table 1) at increased concentration of mucin is much lower compared to all the other investigated compounds (37% at 0.6% mucin concentration; Table 3, Figure 3). The only substantial difference within the drugs investigated (beside their size) is the water solubility at neutral pH. In fact ATN, CAF, and NPR are all sparingly/soluble at this pH (from 2 mg/mL for ATN and NPR to more than 20 mg/mL for CAF, Table 1), whereas HYD is poorly soluble (0.4 mg/mL [18]). That means that ATN, CAF and NPR might be more affected by the presence of mucin (hydrophilic in comparison to HYD). However, further studies should be conducted to shine light on this phenomenon. Moreover, it has been previously demonstrated that the diffusion of HYD in purified gastric mucin type II was comparable to the one in phosphate buffer, whereas in the case of native pig intestinal mucus HYD diffusion was impaired to a higher extent [24,25]. This finding suggests that the study of drug diffusion in mucus should be carried out in the presence of more biorelevant mucus sources. Concerning this, it has been demonstrated that the grade of purification of mucin from porcine stomach has a clear effect on the structure of the mucin mesh, thus affecting the extent of mucin–drug interaction [11]. On the other hand, it can be expected that the drug diffusion from mucin will eventually stop decreasing linearly with increasing mucin concentration, and that it will reach a plateau. This trend has been observed in our previous studies with the mucus–PVPA using increasing concentration of mucin from 1.0 to 4.0% (*w/w*), where no significant change in overall drug permeability was seen [20]. This could explain the behavior of HYD shown in Figure 3, where the diffusion of the drug did not change from MUC 0.3% to MUC 0.6%.

**Figure 4:**
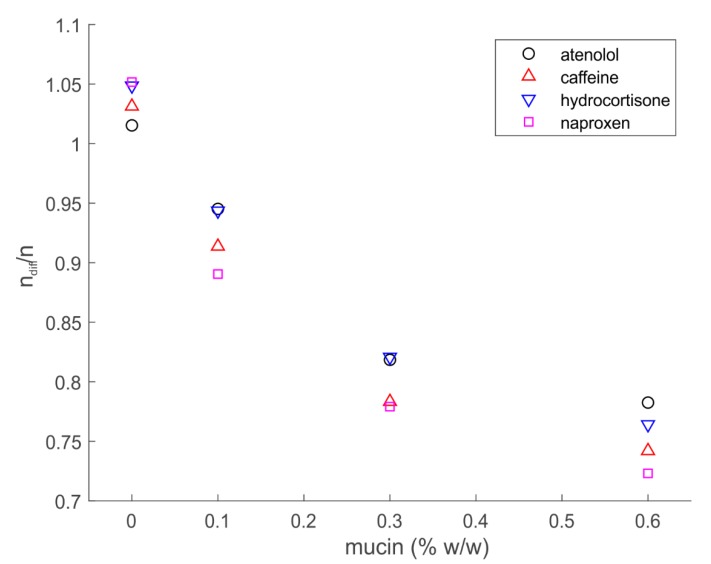
Ratio of drug amount observed (n_dif_) and injected nominal amount (n_i_) as function of mucin concentration.

It should also be noted that the correlation between C_n_ and A in the presence of mucin for all investigated drugs differs from the one in water. In fact, considering a cuvette with cross section (S) of 0.2 cm^2^ and an injection volume (V_i_) of 25 μL, we expect the amount of API in the cuvette during the diffusion experiment ($n_{dif}=SA$) to be the same as the amount of API injected in the cuvette ($n_{i}=V_{i}C_{eq}$, where C_eq._ is the nominal equilibrium concentration). We expect thus that fitting A as a function of C_n_ would yield a straight line with slope V_i_/S. Indeed, this is the case for all APIs in all the different mucin concentrations (*R^2^* ≈ 0.998). However, only in the absence of mucin does the amount of API observed in the diffusion experiments correspond to the amount injected the cuvette. The higher the mucin concentration the more API is not accounted for (see Figure 4), which implies that a certain amount of drug is not available for diffusion in the presence of mucin. This evidence further suggests that interactions are occurring between the drugs and mucin, and shows the value of the current method for the investigation on the impact that mucin has on the diffusion of drugs. These findings seem to imply that not all drugs might be absorbed through a mucosal tissue when in the presence of thick and highly concentrated mucus layers.

### 3.3. Permeability of Drugs In the Presence and Absence of Mucin

The permeability of ATN, CAF, HYD, and NPR was investigated both in the presence and absence of mucin, in order to study the impact of this layer on drug permeation.

**Figure 5:**
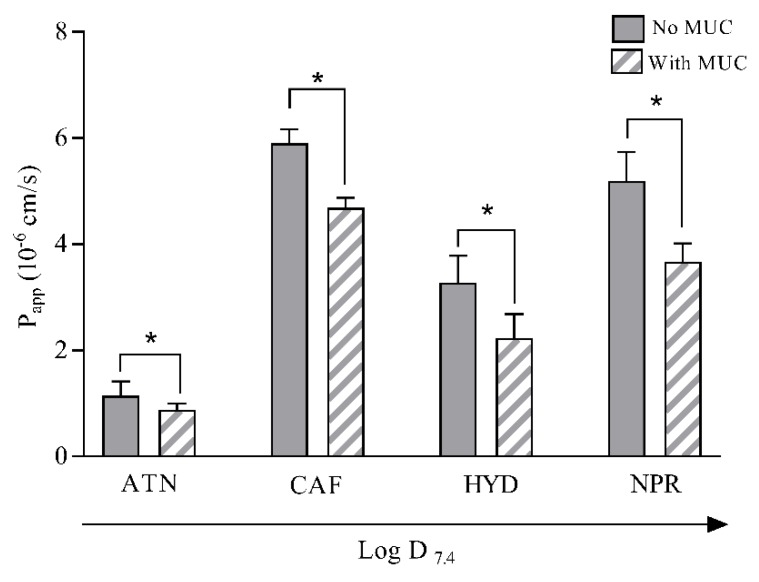
Apparent permeability (P_app_) of CAF, ATN, NPR, and HYD with and without mucin 1.0%. The results are shown as mean ± SD (*n* = 18). * Significant difference (*p* < 0.05) between the presence and absence of mucin (MUC 1.0%).

As observed in Figure 5, the apparent permeability (P_app_) of all four drugs significantly decreased (*p* < 0.05) in the presence of the mucin layer. The change was however found to be to a different extent according to the specific drug, and a trend can be seen showing that permeation of the more hydrophilic drugs ATN and CAF (Table 1) was slowed down in the presence of mucin to a lower extent compared to the more lipophilic ones NPR and HYD (Figure 5). This is in agreement with previous findings where the mucus–PVPA barriers were employed [20,21]. The impact of the mucin layer on the permeation of drugs with different physicochemical characteristics can be traced back to either the interaction or size filtering events described in Section 3.2.

Even though a trend can be observed, the reduction in P_app_ in the presence of mucin was not significantly different between the different drugs. For this reason, the parallel examination of the diffusion from mucin with the use of the UV-visible localized spectroscopy can add useful information regarding the specific interaction of the examined drugs with the mucin layer. In fact, it has to be noted that the investigation discussed in this section takes into account an experimental setup where the diffusion of drugs through the mucin layer is followed by their permeation through the phospholipid-based barriers. This system is therefore more complex than the one discussed in Section 3.2, where the single diffusion of the drug from the mucin is assessed. Thus, for the investigation on the impact of the mucin layer on drug permeation the mucus–PVPA barriers should be used, whereas if the sole drug diffusion through mucin needs to be understood, UV-visible localized spectroscopy should be the favored methodology.

It has to be noted that the mucin concentration used in the diffusion experiment discussed in Section 3.2 (MUC 0.1–0.6% *w/w*) differed from the one used for the assessment of drug permeability in the presence of mucin (1.0% *w/w*). This was due to the fact that we wanted to investigate if very low concentrations of mucin would have an impact on drug diffusion, and that the experimental evaluation of drug diffusion was limited at high mucin concentrations (> 0.6% *w/w*). However, both of these experimental investigations took into account the physiological composition of mucus (where mucin are generally not more than 5% *w/w* [26]). Moreover, it is interesting to notice that the increase in mucin concentration (MUC 0.1–0.6% *w/w*) had an increasing impact on drug diffusion (Figure 3), whereas the same could not be stated regarding drug permeability in our previous work where mucin ranged from 1.0% to 4.0% (*w/w*) [20]. This is most likely due to the fact that at certain mucin concentrations the influence of mucin on drug diffusion/permeation reaches a plateau, potentially due to the effect of viscosity, as discussed in Section 3.2 and showed in Figure 3 in the case of HYD.

### 3.4. Diffusivity–Permeability Correlation

**Figure 6:**
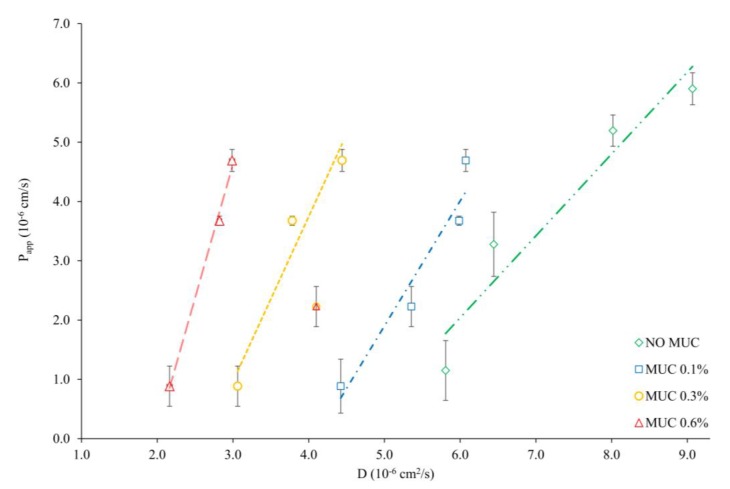
Correlation between apparent permeability (P_app_, average of 18 parallels and relative SD) and diffusivities (D) of CAF, ATN, NPR, and HYD in the absence (green diamonds), and presence of mucin (blue squares, 0.1% MUC; yellow circles, 0.3%; red triangles 0.6% MUC). For correlations at MUC 0.3% and 0.6% the data points related to HYD have not been taken into account.

The correlation between the diffusion (Section 3.2) and permeation (Section 3.3) data was analyzed and displayed in Figure 6. As it can be observed, a linear correlation is found between P_app_ and D_0_ (i.e., in the absence of mucin, *R^2^* = 0.9), which was not observed with other relevant properties such as LogP and LogD _7.4_. This rather remarkable finding points out that the permeability of the four drugs investigated in this study is highly dependent on drug diffusivity in the water layer (unstirred water layer), and less on drug lipophilicity/partitioning properties. Very interestingly, this trend could be observed also at lower mucin concentration (MUC 0.1%) (*R^2^* = 0.9291, Figure 6). On the other hand, the same cannot be found in the presence of mucin at higher concentrations (MUC 0.3% and 0.6% *w/w*). In fact, the coefficient of determination was found to decrease with MUC 0.3% and 0.6% (*R^2^* = 0.6843 for MUC 0.3%; *R^2^* = 0.0424 for MUC 0.6%). In particular, the drug that seemed to be causing this shift was HYD, especially when its diffusion was measured in the presence of 0.3% and 0.6% MUC. Thus, if the results related to HYD MUC 0.3% and 0.6% are not taken into consideration (Figure 6) the linear relationship between P_app_ and D is maintained even in the presence of mucin (*R^2^* = 0.9451 MUC 0.3%; *R^2^* = 0.9952 MUC 0.6%). As discussed in Section 3.2, it has been previously demonstrated that the diffusion of HYD in gastric pig mucin was not hindered compared to phosphate buffer, whereas native pig intestinal mucus proved to slow down the diffusion of this drug [24,25]. This suggests that the diffusivity results related to HYD in this specific experimental setup should be cautiously assessed.

Overall, the correlation depicted in Figure 6, suggests that the permeation of the four investigated drugs is closely related to their diffusion. Moreover, the relation between relative diffusivity and permeability seems to be dependent on the concentration of mucin (Figure 6).

## 4. Conclusions

In the present study, we investigated the applicability of the previously developed UV-visible localized spectroscopy method for the assessment of mucus–drug interactions. The validity of the method was proven by the correspondence between experimental and computational data. The relative diffusivities (D_rel_) of ATN, CAF, and NPR in the presence of mucin depended on the mucin concentration, and the higher the concentration of the mucin, the lower the D_rel_ for all compounds, to different extents. Even though it is not possible to draw definitive conclusions on the selective influence of mucin on drugs according to API chemical space, it is however clear that mucin, especially at higher concentrations (≥ 0.6%), significantly hampers the diffusion of all investigated compounds. This phenomenon can be due to the synergism between the effect that the viscosity of the mucin layer can have on drug diffusion, and to the compound-specific interactions that can occur between mucin and the specific drug. This mucin-effect was observed by both UV-visible localized spectroscopy as well as mucus–PVPA model, supporting the theory of mucin being an additive barrier to permeation. Overall, this method proved to be suitable for the investigation on the extent of interaction occurring between mucin and drugs, allowing for a new, easy and cost-effective investigation of the process affecting mucosal drug administration.

## Figures and Tables

**Table 1:** Physicochemical characteristics of atenolol (ATN), caffeine (CAF), hydrocortisone (HYD,) and naproxen (NPR).

Drug	MW (g/mol)	pKa ^a^	Charge at pH 7.4 ^a^	Log P ^a^	Log D _7.4_^b^	Solubility in Water at pH 7.4 (mg/mL)
ATN	266.34	9.6	+	0.16	−1.03	2.4 ^c^
CAF	194.2	10.4	0	−0.07	−0.07	>20 ^c^
HYD	362.5	12.6	0	1.6	1.37	0.4 ^d^
NPR	230.3	4.2	-	3.2	1.70	3.5 ^e^

^a^: The pKa values, charge at pH 7.4 and LogP were obtained from Drugbank (www.drugbank.ca) [15]; ^b^: The Log D _7.4_ were obtained from Benet et al., 2011 [16]; ^c^: The solubilities were obtained from PubChem (www.pubchem.com) [17]; ^d^: Solubility value obtained from di Cagno and Luppi, 2013 [18]; ^e^: Solubility value obtained from Lam et al., 2019 [19].

**Table 2:** Analytical parameters obtained from Equation (1). C_n_ expresses the nominal initial concentration in the donor, D_0_ the diffusion coefficient in plain water, A_0_ the initial amount injected and σ the width of the initial distribution of the tested drug (ATN, CAF, HYD, and NPR). Data reported are the average of two parallel experiments (variation below 5% and fitting error below 1%).

Drug	C_n_ (mM)	D_0_ (10^−6^ cm^2^/s)	A_0_ (µmol/cm^2^)	σ (cm)
ATN	4.99	5.81	648	0.11
CAF	0.86	9.07	111	0.11
HYD	0.53	6.44	69	0.11
NPR	1.01	8.02	133	0.13

**Table 3:** Percentage reduction in diffusivities (D) with increasing mucin concentrations (0.1–0.6% MUC) compared to the absence of mucin for ATN, CAF, HYD, NPR, and MUC.

MUC%	D Reduction (%)				
	ATN	CAF	HYD	NPR	MUC
0.0	0	0	0	0	0
0.1	24	33	17	25	14
0.3	47	51	36	53	43
0.6	63	67	37	65	39

## Supplementary Materials

The following are available online at https://www.mdpi.com/1999-4923/12/2/168/s1, Figure S1: Diffusion profiles of naproxen (NPR) in water (**A**), MUC 0.1 (**B**), 0.3 (**C**) and 0.6% (**D**) (*w*/*w*). In this figure the drug concentration (C) has been normalized over the nominal concentration of the drug in the cuvette at the beginning of the experiment (C_0_), Figure S2: Diffusion profiles of atenolol (ATN) in water (**A**), MUC 0.1 (**B**), 0.3 (**C**) and 0.6% (**D**) (*w*/*w*). In this figure the drug concentration (C) has been normalized over the nominal concentration of the drug in the cuvette at the beginning of the experiment (C_0_), Figure S3: Diffusion profiles of hydrocortisone (HYD) in water (**A**), MUC 0.1 (**B**), 0.3 (**C**) and 0.6% (**D**) (*w*/*w*). In this figure the drug concentration (C) has been normalized over the nominal concentration of the drug in the cuvette at the beginning of the experiment (C_0_).
