# Supplementary material for: Impact of Mucin on Drug Diffusion: Development of a Straightforward In Vitro Method for the Determination of Drug Diffusivity in the Presence of Mucin
Source: Pharmaceutics. 2020 Feb 17;12(2):168. doi: 10.3390/pharmaceutics12020168 (PMC7076515; doi:10.3390/pharmaceutics12020168)
Supplement: Supplementary file 1 [file pharmaceutics-12-00168-s001.pdf]

pharmaceutics

MDPI

Supplementary Materials: Impact of Mucin on Drug Diffusion: Development of a Straightforward in Vitro Method for the Determination of Drug Diffusivity in the Presence of Mucin

Margherita Falavigna, Paul C. Stein, Gøril Eide Flaten and Massimiliano Pio di Cagno

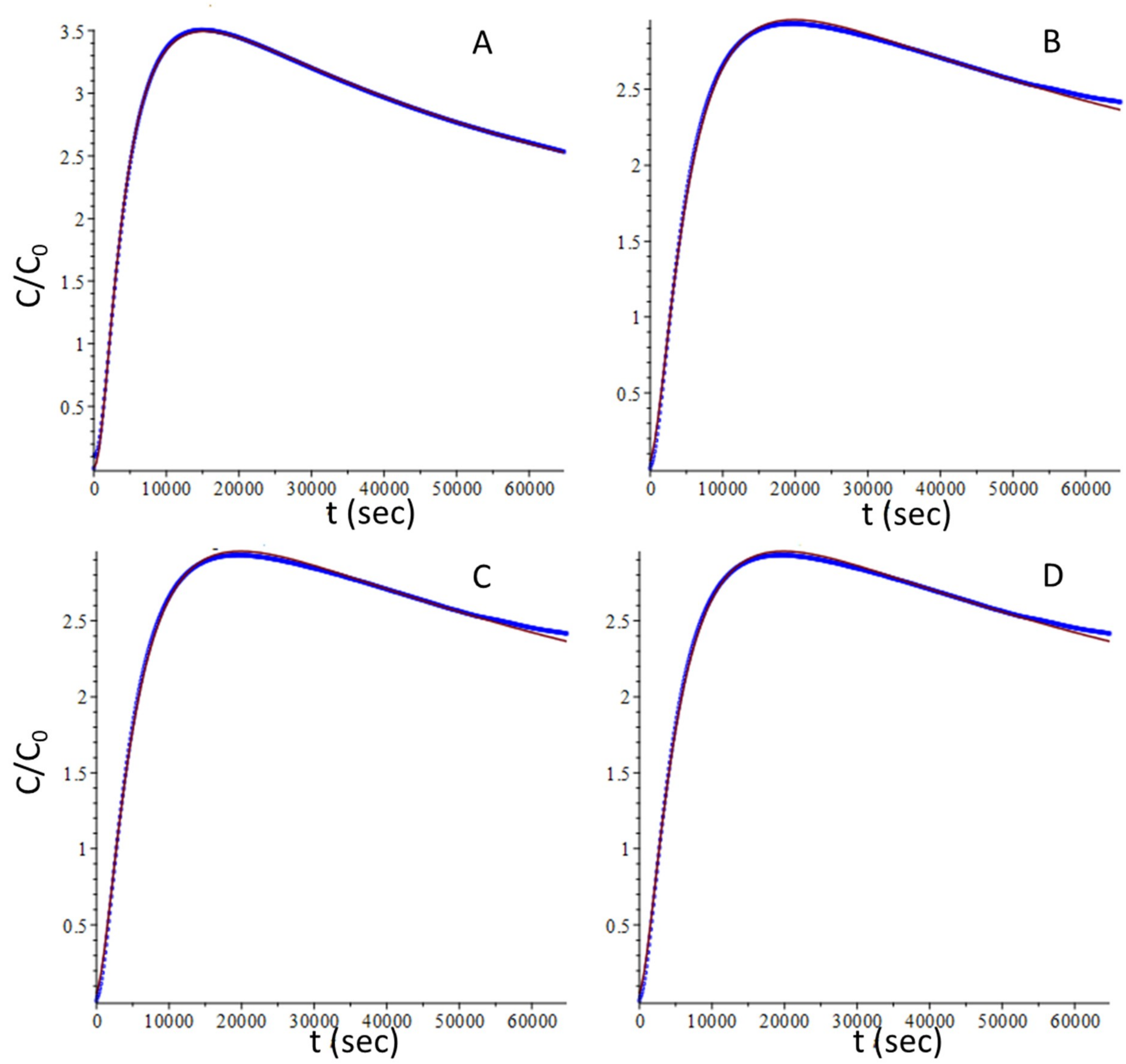

Figure S1. Diffusion profiles of naproxen (NPR) in water (**A**), MUC 0.1 (**B**), 0.3 (**C**) and 0.6% (**D**) (*w/w*). In this figure the drug concentration (C) has been normalized over the nominal concentration of the drug in the cuvette at the beginning of the experiment (C_0).

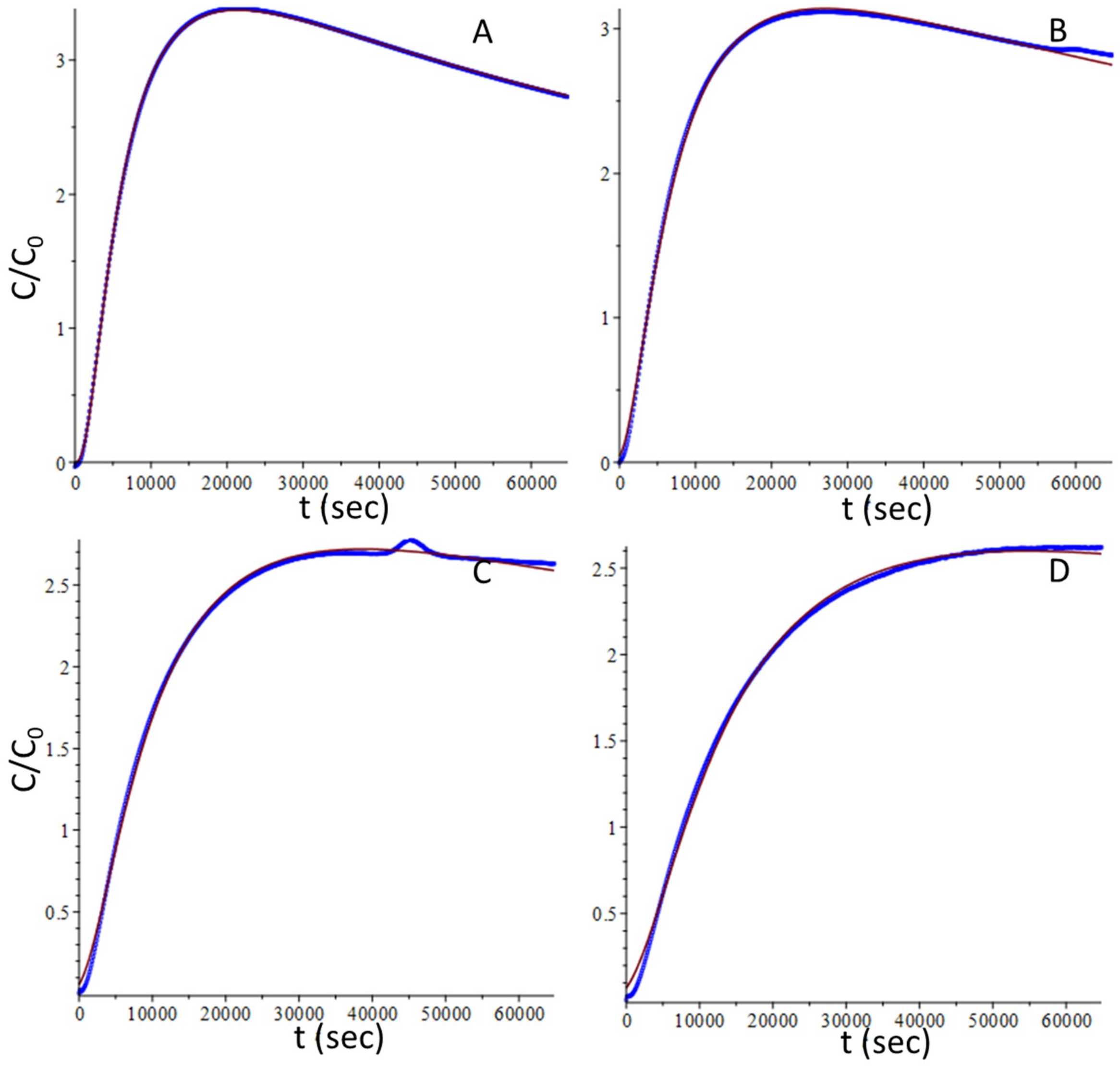

Figure S2. Diffusion profiles of atenolol (ATN) in water (**A**), MUC 0.1 (**B**), 0.3 (**C**) and 0.6% (**D**) (*w*/*w*). In this figure the drug concentration (C) has been normalized over the nominal concentration of the drug in the cuvette at the beginning of the experiment (C_0).

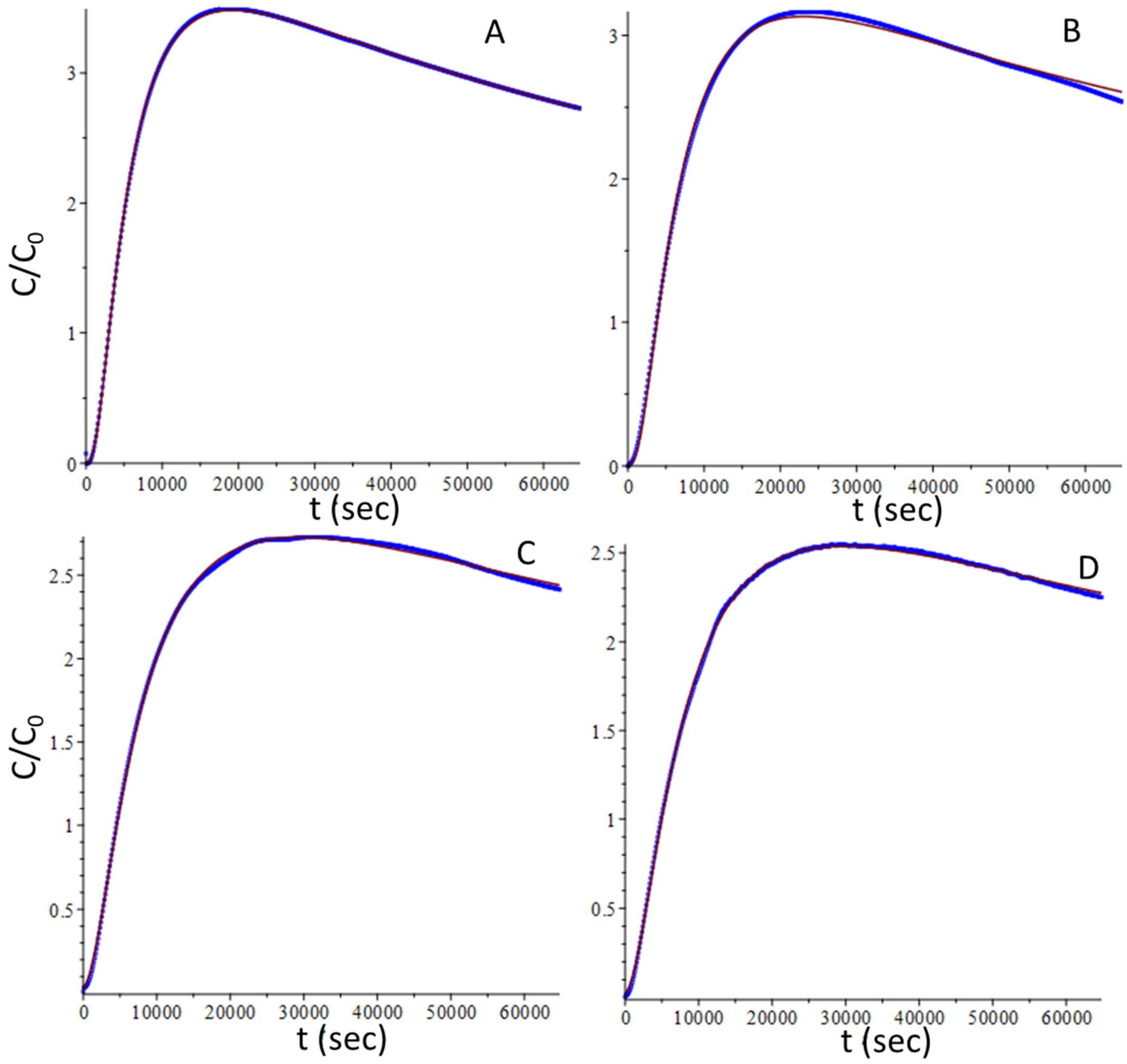

Figure S3. Diffusion profiles of hydrocortisone (HYD) in water (**A**), MUC 0.1 (**B**), 0.3 (**C**) and 0.6% (**D**) (*w/w*). In this figure the drug concentration (C) has been normalized over the nominal concentration of the drug in the cuvette at the beginning of the experiment (C_0).
